# Modulation of small leucine-rich proteoglycans (SLRPs) expression in the mouse uterus by estradiol and progesterone

**DOI:** 10.1186/1477-7827-9-22

**Published:** 2011-02-04

**Authors:** Renato M Salgado, Rodolfo R Favaro, Telma MT Zorn

**Affiliations:** 1 Laboratory of Reproductive and Extracellular Matrix Biology, Department of Cell and Developmental Biology, Institute of Biomedical Sciences, University of São Paulo, São Paulo, Brazil

## Abstract

**Background:**

We have previously demonstrated that four members of the family of small leucine-rich-proteoglycans (SLRPs) of the extracellular matrix (ECM), named decorin, biglycan, lumican and fibromodulin, are deeply remodeled in mouse uterine tissues along the estrous cycle and early pregnancy. It is known that the combined action of estrogen (E2) and progesterone (P4) orchestrates the estrous cycle and prepares the endometrium for pregnancy, modulating synthesis, deposition and degradation of various molecules. Indeed, we showed that versican, another proteoglycan of the ECM, is under hormonal control in the uterine tissues.

**Methods:**

E2 and/or medroxiprogesterone acetate (MPA) were used to demonstrate, by real time PCR and immunoperoxidase staining, respectively, their effects on mRNA expression and protein deposition of these SLRPs, in the uterine tissues.

**Results:**

Decorin and lumican were constitutively expressed and deposited in the ECM in the absence of the ovarian hormones, whereas deposition of biglycan and fibromodulin were abolished from the uterine ECM in the non-treated group. Interestingly, ovariectomy promoted an increase in decorin, lumican and fibromodulin mRNA levels, while biglycan mRNA conspicuously decreased. Hormone replacement with E2 and/or MPA differentially modulates their expression and deposition.

**Conclusions:**

The patterns of expression of these SLRPs in the uterine tissues were found to be hormone-dependent and uterine compartment-related. These results reinforce the existence of subpopulations of endometrial fibroblasts, localized into distinct functional uterine compartments, resembling the organization into basal and functional layers of the human endometrium.

## Background

The reproductive cycle of human and rodents is characterized by recurring morphophysiological changes in the reproductive organs. The combined action of estrogen (E2) and progesterone (P4) orchestrates the cycle and prepares the endometrium for pregnancy. In the mouse, the cycle is known as estrous cycle and is divided into four different phases, denominated proestrus, estrus, metaestrus and diestrus, each one presenting distinct morphological and molecular features [1].

E2 produced during estrus stimulates epithelial cell proliferation and synthesis of progesterone receptors (PR). On the other hand, P4 inhibits epithelial proliferation and stimulates the multiplication of endometrial stromal cells [2,3]. Estrogen receptors (ER) and PR are transcription factors that regulate gene expression by direct binding to DNA regulatory sequences or by specific interactions with co-activators and/or co-repressor proteins [4,5]. It has been previously demonstrated that the uterus of ERα knock-out mice is hypoplastic, the endometrial stroma is disorganized, and the luminal epithelium is formed by cuboidal cells, which are unable to acquire a tall columnar phenotype [6]. PR knock-out mice showed that P4 is a crucial regulator of reproductive functions, as these animals are unable to ovulate, present uterine dysfunction, and altered endothelial and smooth muscle cell proliferation [7]. Moreover, our group showed a clear compartmentalization in the expression of estrogen receptors in the mouse uterine tissues [8].

Among the striking effects promoted by ovarian steroid hormone in the uterine tissues, we emphasize the remodeling of extracellular matrix (ECM) molecules. The ECM is a complex structure of secreted macromolecules, immobilized in the extracellular space, and composed predominantly of collagens, non-collagenous multiadhesive glycoproteins, elastin, hyaluronan and proteoglycans [9].

Decorin, biglycan, lumican and fibromodulin are members of the family of small leucine-rich-proteoglycans (SLRPs) of the ECM [10,11]. The SLRP family comprises about seventeen genes that share structural homologies, such as cysteine residues, leucine rich repeats and at least one glycosaminoglycan side chain. These proteoglycans are divided into five distinct classes. Decorin and biglycan belong to class I, presenting similarities in their amino acid sequence, in the chondroitin or dermatan sulfate side chains and a typical cluster of cysteine residues at the N-terminus that form two disulfide bonds. Fibromodulin and lumican belong to class II, both presenting keratan sulfate and polylactosamine side chains, as well as clusters of tyrosine-sulfate residues at their N-termini [12].

Some SLRPs act as a growth factor reservoir in the ECM, modulating biological processes, such as cell proliferation and differentiation [10,13]. They are capable of inducing signaling cascades through tyrosine kinase, toll-like and TGF-β/BMP receptors [12]. There is strong evidence that collagen fibril-associated decorin is able to arrest TGF-β in the ECM, inhibiting its proliferative activity [14]. Biglycan, on the other hand, has been related to the activity of BMPs in the control of embryo development [15]. In addition, these molecules also participate in the process of collagen fibrillogenesis [10]. The orientation and aggregation of collagen fibrils is partly determined by proteoglycans, as they form interfibrillar bridges [16]. Lumican and fibromodulin, for instance, are known to compete in vitro for the same attachment sites at the collagen fibril, hindering fibrillar lateral growth [13,17,18].

It is known that the endometrial ECM plays important roles in decidualization, embryo implantation and trophoblast cell invasion [19]. For that purpose the endometrium must undergo complex cycles of ECM breakdown and re-arrangement, through coordinated synthesis, deposition and cleavage of its molecules.

Considering the actions of ovarian hormones in the remodeling of uterine tissues, it is reasonable to indicate that proteoglycans are modulated in the uterine ECM by these steroid hormones, whose levels fluctuate constantly in the non-pregnant and pregnant endometrium. Confirming this hypothesis our group has demonstrated that versican expression and deposition are under hormonal control in the mouse uterine tissues [20]. P4 stimulates versican deposition in the endometrium, whereas the myometrium responds exclusively to E2, evidencing that the changes observed in the cellular and ECM organization of the uterine tissues require exposure to a defined hormonal regimen.

We have previously shown the differential distribution of these SLRPs in the mouse endometrium (stroma and epithelia) and myometrium during the estrous cycle [21] and the early stages of pregnancy [22]. In this context, the aim of this study is to characterize the effects of E2 and/or medroxiprogesterone acetate (MPA) treatment on the expression and distribution of decorin, biglycan, lumican and fibromodulin in the uterine tissues of ovariectomized mice.

## Methods

### Animals and tissue collection

Swiss female mice, aged 3-5 months, were used in this study. Animals were housed in a 12-h light: 12-h dark, temperature-controlled (22°C) environment, with free access to food and water. The stages of the estrous cycle were determined by vaginal smears. Animals in estrus and diestrus (physiological control of E2 and P4 levels, respectively), and ovariectomized animals submitted or not to hormone treatment were anesthetized with an intraperitoneal injection of tribromoethanol (Avertin^®^) (Aldrich Chemical Company, Inc., Milwaukee, Wi, USA; 0,025 mL/g body weight). The uteri were subsequently removed, cut with razor blades and immediately immersed in a fixative solution or in RNAlater solution (Sigma-Aldrich, St. Louis, MO, USA). National guidelines for laboratory animal care were followed, and all experiments were approved by the Institute of Biomedical Sciences Animal Ethics Committee (authorization number, 144/2002).

### Ovariectomy and hormone replacement

The general protocol adopted by us [23] was adapted from Domino and Hurd [24]. During standardization of this experimental protocol, the established doses were 10 μg of 17β-estradiol (Sigma-Aldrich) and 0.5 mg of medroxyprogesterone acetate (MPA) (Pharmacia & Upjohn), a progesterone analog. The choice was based on vaginal smear features and uterine morphology, which were similar to what was observed in the cycling mice [21]. Mice were ovariectomized and divided into five experimental groups, as follows:

1. Tissue collection twenty days after ovariectomy without hormone treatment.

2. Pre-treatment with daily priming doses of E2, diluted in mineral oil (Schering-Plough), for three days, followed by a two-day rest and daily injections of E2 during four consecutive days.

3. Pre-treatment with daily priming doses of E2, followed by a resting period and daily injections of MPA, diluted in distilled water, during four consecutive days.

4. Same pre-treatment and resting period as the previous groups, followed by daily injections of both E2 and MPA during four consecutive days.

5. Control group received injections of vehicle alone (mineral oil) for three days, followed by a two-day rest and daily oil injections during four consecutive days.

All injections were sub-cutaneous in a 100 μl volume. Twenty four hours after the last injection, the mice were anesthetized and the uterine samples were collected as described above.

### Light microscopy processing

The samples were fixed at 4°C for 3 h in Methacarn (absolute methanol, chloroform and glacial acetic acid; 6:3:1), rinsed with absolute ethanol, and embedded in Paraplast (Oxford, St. Louis, MO, USA) at 60°C. 5 μm sections were adhered onto glass slides pre-coated with 0.1% poly-L-lysine (Sigma, St. Louis, MO, USA) and then dried at 37°C.

### Immunoperoxidase procedure

The immunoperoxidase staining was performed as previously described [21]. Sections were treated with 3% (v/v) H_2_O_2 _in PBS (30 min) to block endogenous peroxidase activity. Each of the succeeding steps was followed by a thorough rinse in PBS. All steps were performed in a humidified chamber. Samples were pretreated with Chondroitinase ABC from *Proteus vulgaris *(Seikagaku, Tokyo, Japan), diluted in 20 mM pH 6,0 Tris-HCl buffer (1 h at 37°C). Nonspecific staining was blocked by incubating the sections for 1 h with normal rabbit serum (for lumican) or goat serum (for the other molecules), diluted 1:1 (v/v) in PBS - 10% BSA (w/v) (room temperature). Sections were then incubated with primary antibodies (Table 1) diluted in PBS containing 0.3% (v/v) Tween 20, overnight (4°C). After extensive rinsing in PBS all sections were incubated for 1 h at room temperature with the specific biotin-conjugated secondary antibody (Table 1) diluted in PBS, for 1 h at room temperature. After rinsing in PBS, sections were incubated with Vectastain ABC kit (Vector Laboratories, Burlingame, CA, USA) for 1 h at room temperature. The peroxidase reaction was visualized using 0.03% (w/v) 3,3'-diaminobenzidine in PBS with 0.03% (v/v) H_2_O_2_. In order to achieve standardization of the immunoreactions, for each antibody, the slides were simultaneously incubated with DAB, and reaction was immediately interrupted with PBS after a specific period of time (1-5 minutes, depending on the antibody). Afterwards, sections were lightly counterstained with Mayer's haematoxilin (Merck, Darmstadt, Germany). For each immunohistochemical reaction, control reactions were performed by omitting the primary antibody step from the protocol. In addition, paraffin sections of mouse embryos were used as positive and negative control.

**Table 1:** Antibodies

Primary Ab	[ ]	Second Ab - Biotin Conjugated (Rockland§)	[ ]
Anti-decorin (LF-113∞)	1:3000	Anti-rabbit (goat)	1:2000
Anti-biglycan (LF-159∞)	1:1000	Anti-rabbit (goat)	1:2000
Anti-lumican (R&D Systems¥)	1:1500	Anti-goat (rabbit)	1:2000
Anti-fibromodulin (LF-150∞)	1:400	Anti-rabbit (goat)	1:2000

∞ Dr. Larry Fisher (National Institute of Dental and Craniofacial Research, NIH, Bethesda, MD)

¥ R&D Systems - Minneapolis, MN, USA

§ Rockland - Gilbertsville, PA, USA

The immunostained sections were examined in a Nikon Eclipse E600 microscope and the images were captured using a digital camera (Cool SNAP-Procf color; Roper Scientific, Trenton, NJ, USA) and Image Pro Plus software (Media Cybernetics, Silver Spring, MD, USA).

### Antibodies

Table 1 lists the antibodies used in the present study. Decorin, biglycan and fibromodulin antibodies (LF-113, LF-159 and LF-150, respectively) were raised in rabbit and recognize the core protein of each macromolecule. These antibodies were previously tested by Western blot by the producers. Detail of the procedures made by Larry Fisher (National Institute of Dental and Craniofacial Research, NIH, Bethesda, USA) may be found in Fisher et al [23].

The anti-mouse lumican antibody (R&D Systems, #AF2745) is an IgG produced in goats, immunized with purified, NS0-derived, recombinant mouse Lumican (rmLumican). The mouse lumican specific IgG was purified by affinity chromatography. The specificity was tested by direct ELISA and Western blot.

### mRNA extraction and real-time PCR

For the molecular biology experiments, the phases of estrus (highest estrogen levels) and diestrus (highest progesterone levels) were chosen to represent the estrous cycle.

Upon use, uterine samples (n = 6 per group), excised with sterilized razor blades and stored in RNAlater solution at -20°C, were transferred to sterile microfuge tubes containing ceramic beads and 500 μl of Trizol reagent (Invitrogen, Calbard, CA, USA). Samples were homogenized and total RNA extracted using the Precellys 24 homogenizer (Bertin Technologies, Saint-Quentin-en-Yvelines, France), following the manufacturer's instructions. RNA quantity and quality were assessed with a NanoDrop spectrophotometer (Thermo Fisher Scientific Inc., USA). Reverse transcription (RT-PCR) was performed with AffinityScript QPCR cDNA Synthesis kit (Stratagene, Cedar Creek, TX, USA) and 1 μg of total RNA. Different reference genes were tested and Ywhaz (*Tyrosine 3-monooxygenase/tryptophan 5-monooxygenase activation protein, zeta polypeptide*) was chosen as internal control for showing the most uniform expression across groups in the PCR amplification experiments. The relative expression of the SLRPs mRNA was determined as described previously [25]. The relative levels of mRNA of the tested genes were estimated in duplicate samples by fluorescence quantified with the ABI Prism 7500 sequence detector (Applied Biosystems, Ontario, Canada). Reactions were performed in a total volume of 25 μl containing 20 ng of cDNA and 450 nM primers in a reaction buffer containing SYBR Green PCR master mix (Stratagene). All *C*_q _(quantification cycle, according to the MIQE guidelines [26]) values were normalized to the expression of the reference gene and the results were expressed as fold-induction relative to the expression of the calibrator sample (estrus), arbitrarily set to 1. Primers were designed with NCBI's primer designing tool to span an exon-exon junction, in order to limit amplification only to mRNA. Primer sequences are given in Table 2.

**Table 2:** Mouse oligonucleotide primers (5'→3')

Gene	Forward (F) and Reverse (R) primers	Amplicon size	Genbank #
Decorin	F: TGCGATCCCTCAAGGTCTGCCT R: TTGGCCAGACTGCCATTCTCCA	162 bp	NM_007833.4
Biglycan	F: AGGAGGCTTCAGGTTCAG R: TAGCAGTGTGGTGTCAGG	171 bp	NM_007542
Lumican	F: CATTAGTCGGTAGTGTCAGTG R: TGCCAGGAGGAACCATTG	171 bp	NM_008524
Fibromodulin	F: TGCCACATTCTCCAACCCAAGG R: GAGCAGAGCCCAGCCAGCAG	116 bp	NM_021355.3
Ywhaz	F: GAAGCCACAATGTTCTTGGCCCAT R: AAACCAACAGAGACTTGGAAGCAC	84 bp	NM_011740.2

### Statistical analysis

Multiple comparisons were performed by one-way analysis of variance (ANOVA) followed by the Student Newman Keuls post-test to determine significant differences among all groups, using Prism 4.0 (GraphPad Software, San Diego, CA, USA). All results are expressed as the mean ± standard error of the mean (SEM). Values of p less than or equal to 0.05 were considered statistically significant.

## Results

Two morphologically distinct compartments can be identified in the endometrial stroma, herein denoted as superficial and deep stroma; in the myometrium, three distinct layers are observed, denominated internal muscle layer, external muscle layer and connective tissue between layers or vascular plexus [21,20].

### Decorin immunolocalization

**Figure 1:**
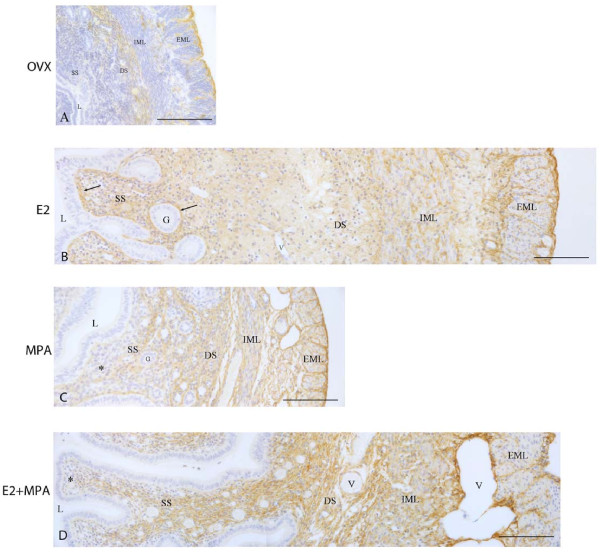
**Immunoperoxidase for decorin**. (A) Ovx group: decorin is present in the deep stroma, the external muscle layer and the connective tissue between layers; (B) E2-group: the immunoreaction is seen in the whole endometrial stroma, especially around glands and under the luminal epithelium (arrows), as well as in both layers of the myometrium; (C) MPA-group: the reaction is observed in the deep stroma and only traces are seen in the superficial stroma (asterisk). In the myometrium, the reaction is present in both layers and the connective tissue; (D) E2+MPA-group: the deposition of decorin is observed in the whole endometrial stroma and in both layers of the myometrium, as well ass in the connective tissue between them. L = uterine lumen; G = endometrial gland; SS = superficial stroma; DS = deep stroma; V = blood vessel; IML = internal muscle layer; EML = external muscle layer. Scale bar: 50 μm.

Ovariectomy did not suppress the deposition of decorin in the uterine tissues. In the non-treated groups (ovx and oil), the immunoreaction was present in the deep stroma and only traces were observed in the superficial stroma. In the myometrium, the immunoreaction was present in the external muscle layer (EML) and connective tissue between layers, but was not observed in the ECM of the internal muscle layer (IML) (Figure 1A).

In the E2-treated group, strong immunoreaction was present in the whole endometrial stroma, observed as a network of thin brownish filaments. The immunoreaction was more intense around the luminal and glandular epithelia, as well as around blood vessels. The staining was also strong in the myometrium, especially around bundles of smooth muscle cells in the EML (Figure 1B).

In the MPA-treated group, the immunoreaction was strong in the deep stroma, however only traces were observed in the subepithelial stroma, as previously observed in diestrus [20]. In the myometrium, the reaction was present in the ECM and inside muscle cells of both layers, and in the connective tissue between layers (Figure 1C).

In the E2+MPA-treated group, the immunoreaction was present in the whole stroma, as a network of thin filaments, except in the subepithelial stroma at the cleft regions of the luminal epithelium branches. In the myometrium, the immunostaining was moderate in the ECM of the IML and strong in the EML and connective tissue between layers (Figure 1D).

### Relative expression of decorin mRNA

**Figure 2:**
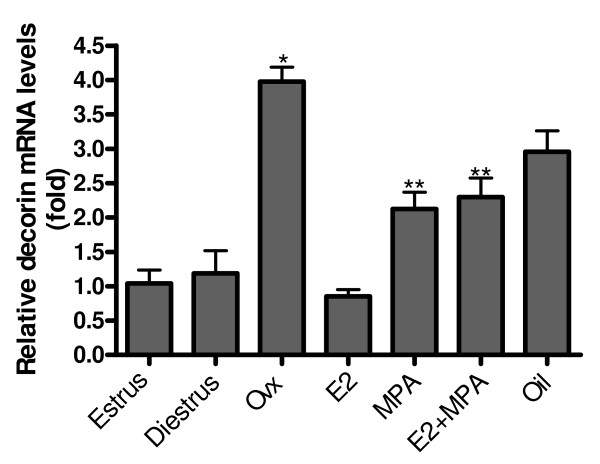
**Decorin mRNA expression**. Ovariectomy (ovx) significantly increases decorin mRNA expression (~4 fold) when compared with estrus. Hormone replacement promotes a significant reduction in decorin expression in the E2-treated group (~4 fold) and MPA and E2+MPA-treated groups (~2 fold). Values represent the mean ± SEM. *p < 0.05; **p < 0.01; ***p < 0.001.

During the estrous cycle, there were no significant differences between groups. Ovariectomy significantly increased decorin mRNA expression (≈4 fold) (p < 0.001) when compared to estrus. Hormone replacement promoted a significant reduction in decorin expression in the E2-treated group (≈4 fold) (p < 0.001) and MPA and E2+MPA-treated groups (≈2 fold) (p < 0.01), when compared to the non-treated ovariectomized group. Moreover, decorin expression in the MPA and E2+MPA-treated groups was significantly higher than in diestrus and estrus, respectively (≈2 fold) (p < 0.01) (Figure 2).

### Biglycan immunolocalization

**Figure 3:**
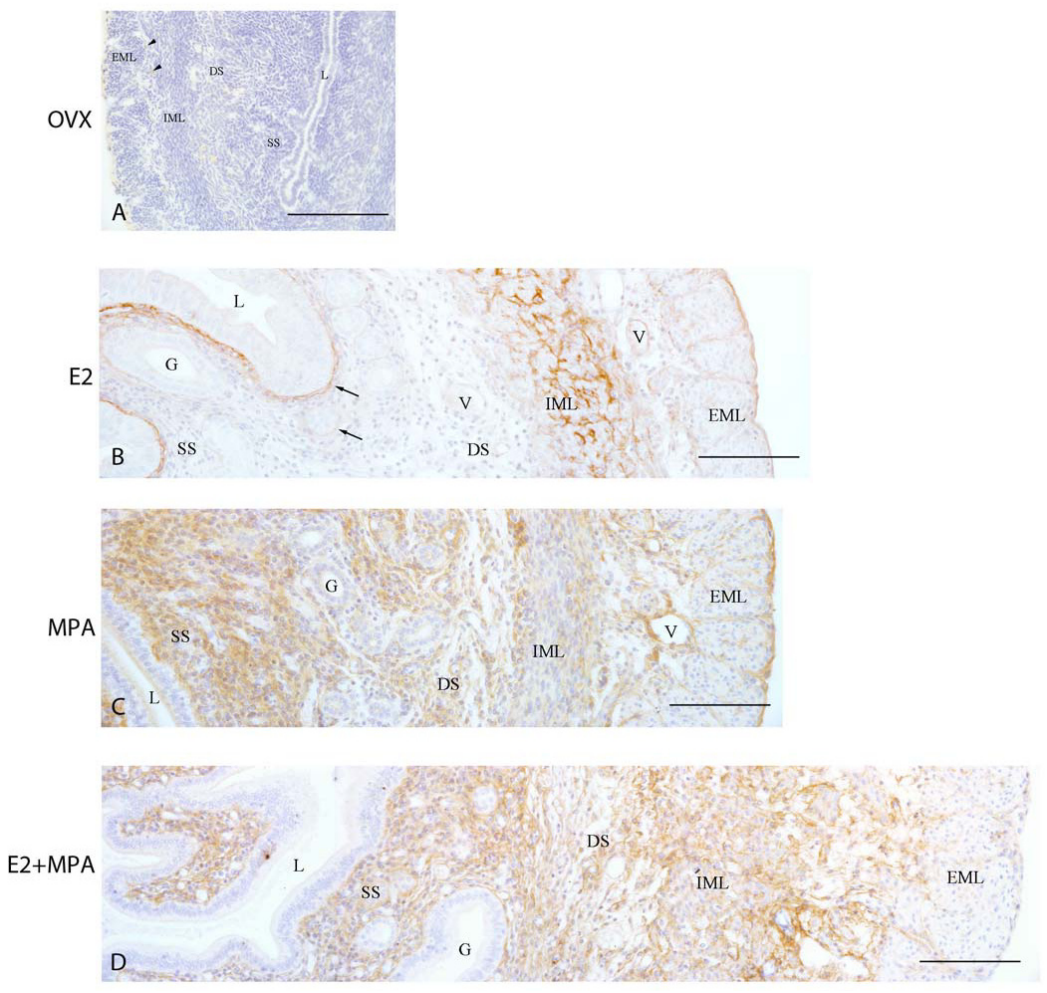
**Immunoperoxidase for biglycan**. (A) Ovx group: the immunoreaction is absent in all uterine tissues, being detected only in the cytoplasm of some leukocytes (arrowhead); (B) E2-group: the reaction is observed in the region of basement membrane of the luminal and glandular epithelia (arrows) and in the myometrium; (C) MPA-group: strong diffuse reaction in the whole endometrial stroma. Moderate immunoreaction is present in both layers of the myometrium and the connective tissue between them; (D) E2+MPA-group: the immunoreaction is distributed as a network of thin filaments in the whole stroma. In the myometrium, the deposition of biglycan is observed in both layers and connective tissue, being weak in the EML. L = uterine lumen; G = endometrial gland; SS = superficial stroma; DS = deep stroma; V = blood vessel; IML = internal muscle layer; EML = external muscle layer. Scale bar: 50 μm.

In the ovx and oil groups, the deposition of biglycan was not observed in the endometrium and myometrium. However, the immunoreaction was detected in the cytoplasm of mononucleated leukocytes (Figure 3A).

In the E2-treated group, the immunoreaction was present in the endometrial stroma mainly around the luminal and glandular epithelia. In the myometrium, the staining was observed in the ECM of both smooth muscle layers, especially the IML, and in the connective tissue between them, particularly around blood vessels (Figure 3B).

In the MPA-treated group, a diffuse brownish staining was observed in the endometrial stroma. In the myometrium, the immunoreaction was moderate in both layers. It was also present in the connective tissue between layers, especially around blood vessels (Figure 3C).

In the E2+MPA-treated group, biglycan was distributed as a network of thin brownish filaments, especially in the subepithelial stroma, where leukocytic infiltration was observed. In the myometrium, the immunoreaction was more conspicuous in the IML and connective tissue between layers, whereas a weak staining was observed in the EML (Figure 3D).

### Relative expression of biglycan mRNA

**Figure 4:**
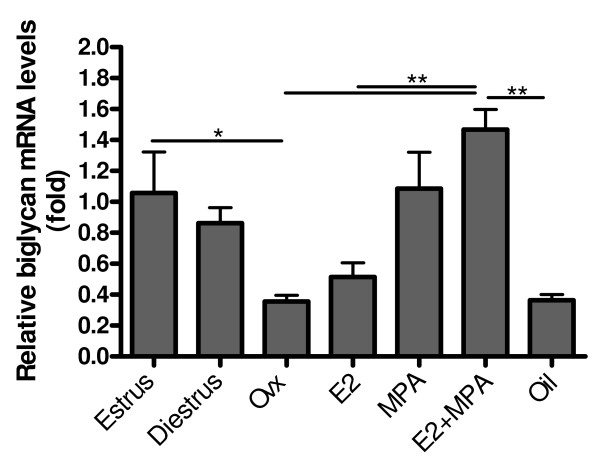
**Biglycan mRNA expression**. After ovariectomy there is a drastic reduction in biglycan expression (~3 fold), when compared with estrus. A significant increase is observed after hormone treatment with MPA (~3 fold) and E2+MPA (~5 fold). Values represent the mean ± SEM. *p < 0.05; **p < 0.01; ***p < 0.001.

As observed for decorin, there was no significant difference in biglycan mRNA levels between estrus and diestrus. However, after ovariectomy there was a drastic reduction in biglycan expression (≈3 fold) (p < 0.001), when compared to estrus. A significant increase was observed after hormone treatment with MPA (≈3 fold) (p < 0.05) and E2+MPA (≈5 fold) (p < 0.001), when compared to the non-treated ovariectomized group. Treatment with E2 alone did not promote significant differences in biglycan mRNA levels in relation to the ovariectomized group. However, it was significantly lower than in estrus (≈2 fold) (p < 0.05) (Figure 4).

### Lumican immunolocalization

**Figure 5:**
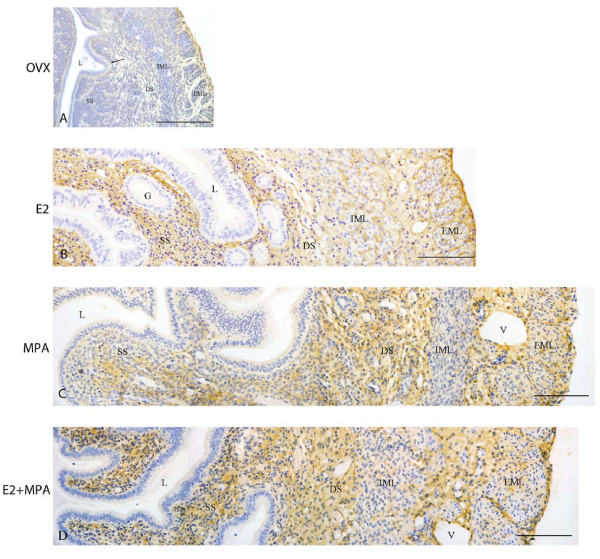
**Immunoperoxidase for lumican**. (A) Ovx group: A weak immunoreaction is observed in the endometrial stroma, especially under the luminal epithelium (arrow) and in the deep stroma, as well as in the EML and connective tissue between muscle layers of the myometrium; (B) E2-group: the reaction is present in the whole endometrial stroma and in both layers of the myometrium; (C) MPA-group: the reaction is present in the whole stroma, however it is weak in the subepithelial stroma (asterisk). In the myometrium, deposition of lumican is observed in the EML and connective tissue between layers; (D) E2+MPA-group: the immunoreaction is strong in the whole endometrial stroma. In the myometrium, a weak staining is observed in both IML and EML, being strong in the connective tissue between them. L = uterine lumen; G = endometrial gland; SS = superficial stroma; DS = deep stroma; V = blood vessel; IML = internal muscle layer; EML = external muscle layer. Scale bar: 50 μm.

In the ovx and oil groups, a faint immunoreaction was observed in the scarce extracellular spaces of the endometrial stroma, especially in the region of basement membrane of the luminal epithelium, as well as in the deep stroma. In the myometrium, the deposition of lumican was weak in the connective tissue between layers, in the EML and in the outer lining of the uterus, the mesothelium (Figure 5A).

In the E2-treated group, lumican was widely distributed in the whole endometrial stroma, in both layers of the myometrium, around bundles of smooth muscle cells, and in the connective tissue between layers (Figure 5B).

In the MPA-treated group, the immunoreaction was present in the whole endometrial stroma as a diffuse brownish staining, particularly in the deep stroma. Under the luminal epithelium, only traces of immunoreaction were observed. In the myometrium, the immunostaining was present only in the EML and connective tissue between layers (Figure 5C).

In the E2+MPA-treated group, strong immunoreaction was present in the whole endometrial stroma. In the myometrium, the immunodeposition of lumican was observed in the connective tissue between layers and a weak staining was present in both myometrial layers, especially in the EML (Figure 5D).

### Relative expression of lumican mRNA

**Figure 6:**
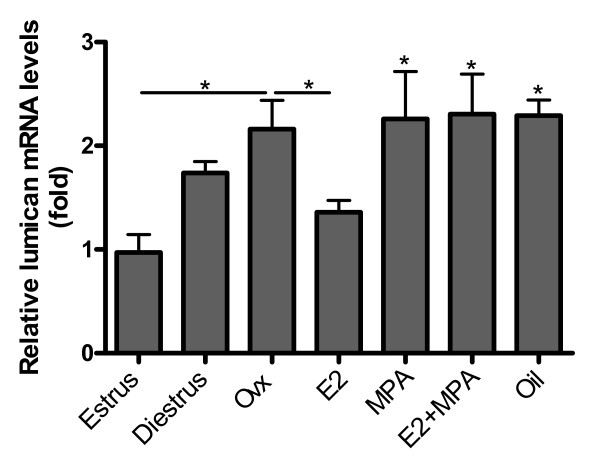
**Lumican mRNA expression**. After ovariectomy there is a ~2 fold increase in lumican mRNA levels, when compared with estrus. Hormone treatments do not significantly alter lumican expression in the uterus. Values represent the mean ± SEM. *p < 0.05; **p < 0.01; ***p < 0.001.

There were no significant differences in lumican expression between estrus and diestrus. After ovariectomy there was a ≈2 fold increase in lumican mRNA levels when compared to estrus. Hormone treatments did not significantly alter lumican expression in the uterus, when compared to the non-treated ovariectomized group, maintaining a ≈2 fold increase in relation to estrus (p < 0.05) (Figure 6).

### Fibromodulin immunolocalization

**Figure 7:**
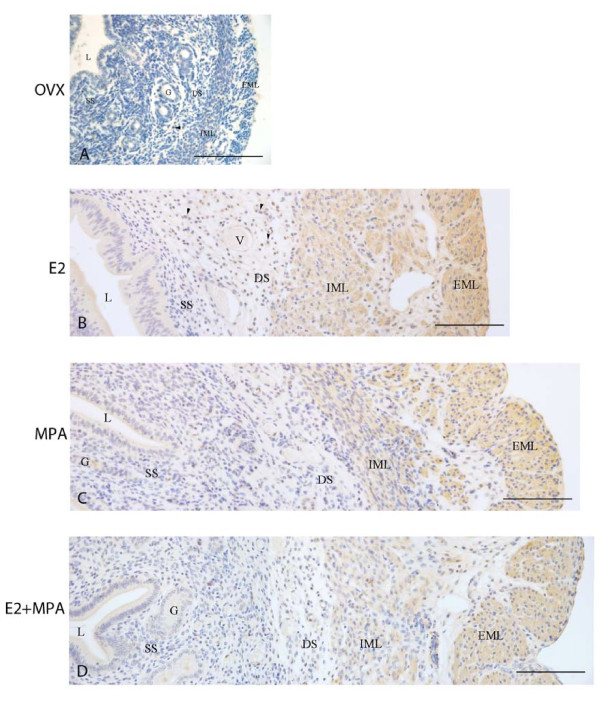
**Immunoperoxidase for fibromodulin**. (A) Ovx group: the immunodeposition is absent from all uterine tissues, but may be observed in the cytoplasm of some leukocytes (arrowhead); (B) E2-group: the immunoreaction is present in the apical cytoplasm of epithelial cells and in leukocytes observed in the stroma (arrowheads). A strong immunoreaction is seen in both layers of the myometrium and in the connective tissue between them; (C) MPA-group: the reaction is present in the luminal epithelium and in the secretion of endometrial glands. The immunoreaction is seen in both layers of the myometrium, especially the EML; (D) E2+MPA-group: the staining pattern is similar to that observed in the MPA group. L = uterine lumen; G = endometrial gland; SS = superficial stroma; DS = deep stroma; V = blood vessel; IML = internal muscle layer; EML = external muscle layer. Scale bar: 50 μm.

In the ovx and oil groups, fibromodulin immunostaining was not observed in the ECM of the endometrium and myometrium. However, some mononucleated leukocytes were immunoreactive in the endometrial stroma, as previously observed during the estrous cycle (Figure 7A).

In the E2-treated group, the immunoreaction was present in the apical region of the luminal epithelium and in the cytoplasm of mononucleated leukocytes observed in the endometrial stroma. Strong immunostaining was present in both myometrial layers, and around blood vessels of the connective tissue between layers (Figure 7B).

In the MPA-treated group, the immunoreaction was present in the luminal epithelium. The glandular secretion was also immunoreactive to fibromodulin. In the myometrium, the immunoreaction was strong in both muscle layers, particularly the EML (Figure 7C).

In the E2+MPA-treated group, the staining pattern was similar to that observed in the MPA group (Figure 7D).

### Relative expression of fibromodulin mRNA

**Figure 8:**
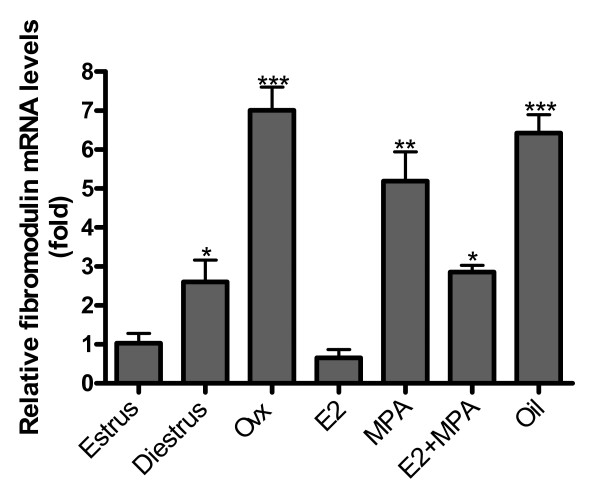
**Fibromodulin mRNA expression**. The expression is significantly higher in diestrus than in estrus (~2 fold). Moreover, ovariectomy promotes a conspicuous increase in fibromodulin mRNA levels, when compared to estrus (~7 fold). Hormone treatments differentially induce decrease in fibromodulin expression (E2 - ~7 fold; MPA - ~2 fold; E2+MPA - ~2.5 fold). Values represent the mean ± SEM. *p < 0.05; **p < 0.01; ***p < 0.001.

Fibromodulin mRNA expression was significantly higher in diestrus than in estrus (≈2 fold) (p < 0.05). Ovariectomy promoted a conspicuous increase in fibromodulin mRNA levels, when compared to estrus (≈7 fold) (p < 0.001). In addition, fibromodulin expression was significantly reduced after E2 treatment (≈7.5 fold) (p < 0.001), when compared to the non-treated ovariectomized group. Moreover, MPA and E2+MPA groups showed significantly augmented expression when compared to diestrus (≈2 fold) (p < 0.01) and estrus (≈2.5 fold) (p < 0.05), respectively (Figure 8).

## Discussion

It is well established that in rodents the endometrium provides a unique environment, which allows or prevents embryo implantation. It is also known that the endometrium undergoes dynamic reorganization during the estrous cycle in preparation for these important events [27]. Concerning the extracellular matrix, previous studies from our group described that the structure and composition of ECM molecules in the distinct uterine tissues is affected throughout the phases of the estrous cycle [21,20,28].

In this study, the expression and distribution of decorin, biglycan, lumican and fibromodulin in the endometrium and myometrium were found to be ovarian hormone-dependent and dose-related, evidencing that those molecules play important roles in the preparation for embryo implantation and successful gestation. Previously, San Martin et al [22] showed that decorin and lumican are present in the superficial stroma on day four of pregnancy, before decidualization. However, decorin disappears from the endometrial stroma, following a progesterone peak and the establishment of the decidua. Interestingly, decorin is absent and lumican deposition is reduced in the superficial stroma in the diestrus phase [21], characterized by high levels of progesterone [29]. Coherently, the present results showed a similar distribution pattern after MPA treatment.

The loss of decorin and reduction of lumican in this context is functionally relevant, as both have anti-proliferative activity [30,31], and in the beginning of decidualization there is considerable DNA synthesis and cell proliferation under strict hormonal control [32,33]. Moreover, decorin is known for its tumor-suppressive role. Hu et al [34] demonstrated that decorin is able to suppress prostate tumor growth through inhibition of the EGFR signaling pathway. Additionally, Gu et al [35] showed that decorin gene expression is down-regulated in mammary tumor tissue when compared to normal mammary gland tissue. Thus, our data suggest that P4 is capable of inhibiting or reducing, directly or through other regulatory molecules, the expression and deposition of decorin in the superficial stroma, while stimulating the opposite effect in the deep stroma, which corroborate high cell proliferation rate in the superficial stroma (unpublished data) and the existence of two genetically distinct subpopulations of endometrial fibroblasts.

This proliferative phenomenon modulated by ECM molecules is important for the preparation of the superficial stroma to decidualize, while maintaining an undifferentiated layer of endometrial fibroblasts in the deep stroma, which is associated with reconstruction of the endometrium in the post-partum period [36]. In addition, these results reinforce the existence of distinct functional compartments of the mouse endometrium, similar to the organization into basal and functional layers of the human endometrium.

Moreover, the SLRPs studied herein are known to regulate collagen fibrillogenesis. Decorin is considered a regulatory molecule, delaying the thickening of collagen fibrils [17,37]. This study from Vogel and Trotter [37] demonstrated that decorin, as well as lumican, link to fibrillar collagens *in vitro*, stabilizing thin fibrils. Ameye et al [38] showed that fibromodulin-deficient mice synthesize abnormal and less numerous fibril bundles, and these fibrils are often irregular and their diameter is decreased. Decorin knock-out mice present irregular fibrils with increased diameter in the endometrial stroma of the pregnant mouse uterus. In addition, there is an augment in lumican deposition in the endometrial ECM of decorin-deficient mice, when compared to wild-type littermate, demonstrating that both SLRPs compensate each other's expression in abnormal situations [39]. During early pregnancy in wild-type mice, the loss of decorin, reduction of lumican and the deposition of biglycan in the decidual ECM are related to the appearance of thick collagen fibrils in the mature and pre-decidua [21]. The only reports on the presence of lumican in the uterus are part of previous studies from our group [21,22,39] and the influence of ovarian hormones on its expression in the uterus was uncertain until now. It is known that lumican knock-out mice are viable, but present reduced body weight and their litter are considerably smaller, suggesting the relevance of this proteoglycan for proper fetal growth [40].

A recent report by Markiewicz et al. [41] describes a significant decrease in lumican, decorin and fibromodulin mRNA levels in the skin of ovariectomized mice, when compared to wild-type littermate. Contrarily, our results showed that withdrawal of ovarian hormones through ovariectomy promotes an augment in decorin, lumican and fibromodulin mRNA expression in the mouse uterus, and that E2 replacement induces a significant decrease in their mRNA levels. Indeed, microarray data has previously demonstrated that decorin and fibromodulin expression is down-regulated by E2 treatment in the uterus of ovariectomized mice, corroborating our findings [42].

Despite the lack of deposition of both biglycan and fibromodulin in the ECM of the endometrium and myometrium in the ovx group, it is clear that withdrawal of ovarian hormones promotes opposite effects on their mRNA expression. Biglycan mRNA levels decreased, whereas fibromodulin mRNA levels conspicuously increased, suggesting that distinct post-transcriptional modifications may occur among closely related molecules. In fact, mature mRNA transcripts may be modified by different reactions, such as alternative transcription start sites, alternative splicing and alternative polyadenylation site selection [43].

In the human myometrium, fibromodulin expression is significantly higher in the secretory phase (progesterone dominance) than in the proliferative phase (estrogenic dominance) of the menstrual cycle [44]. We demonstrated that, after hormone treatments, fibromodulin deposition was strong in the myometrial ECM, as observed during the estrous cycle and the early stages of pregnancy, which suggests that fibromodulin acts in a hormone-dependent manner in the remodeling of the myometrium. The present findings also showed that deposition of all four SLRPs was abolished from the internal and external layers of the myometrium after ovariectomy, evidencing that this uterine compartment is highly sensitive to fluctuations in ovarian hormone levels.

Curiously, only biglycan and fibromodulin were detected in the cytoplasm of uterine epithelial cells during the estrous cycle and after hormone replacement. Schaefer et al [45] showed the deposition of biglycan in glomerular endothelial cells of renal tubules, whereas Qian et al [46] demonstrated that fibromodulin is expressed in gingival epithelium, indicating that epithelial cells synthesize and secrete those proteoglycans in the ECM and/or act in tissue remodeling through internalization of secreted molecules. Biglycan is a well-known proteoglycan of pericellular matrices [12] and modulates cell signaling events. Only in the E2-treated group, biglycan was deposited preferentially in the region of epithelial basement membrane and around blood vessels, suggesting that its deposition is regulated by E2 in a dose-dependent manner.

Hormone actions on target cells involve complex molecular mechanisms. In our model, this complexity may be exacerbated through the activity of metalloproteinases and growth factors, such as TGF-β. The glycosaminoglycan side chains attached to the proteoglycan molecule possibly create a permissive environment for the accumulation of growth factors in the ECM, thus modulating cell metabolism.

Studies by Kim at al [47] showed that, in women, after hormonal stimulus by E2 and P4, TGFβ expression is up-regulated in the endometrium, being down-regulated after P4 withdrawal. Unpublished data from our group showed that TGFβ is widely distributed in the mouse endometrial stroma in the phases of estrus and diestrus. A previous report showed that TGFβ 1, 2 and 3 are expressed in mouse uterine tissues during early pregnancy [48]. Additionally, P4 is capable of inducing the expression of TGFβ1 by uterine epithelial cells and stromal fibroblasts [47]. It is known that SLRPs modulate TGFβ activity in the uterine stroma and other tissues, controlling important biological processes, such as cell proliferation and apoptosis [12,49]. It should be considered, however, that the regulation of cellular responses to growth factors depends not only on the presence, but also on the concentration of these molecules in the tissues.

Additionally, it is important to acknowledge the role of specific proteases in this modulation process. For instance, a previous report by Monfort et al [50] described that decorin, lumican, biglycan and fibromodulin are cleaved by metalloproteinase 13 (MMP-13) in cartilage, at specific aminoacid sequences and at distinct rates. Imai et al [51] showed that decorin is cleaved, at different sites, by MMPs 2, 7 and especially MMP 3, a well known proteoglycanase of many tissues. The controlled cleavage of decorin is related to TGF-β release in the ECM. Thus, MMPs may exert distinct actions under specific hormonal influence. These molecules may also generate bioactive peptides that play key roles in tissue remodeling. Indeed, versican, an aggregating proteoglycan widely distributed in the mouse endometrium, is cleaved by ADAMTS, generating specific peptides that most likely participate in biological processes, such as cell migration, proliferation, and differentiation [52].

Another important variable in the context of steroid hormone action is the regulation of mRNA stability of several molecules, as well as the activity of RNases [53-55]. Small RNA species, such as microRNAs and small interference RNAs, are known to regulate gene expression in a posttranscriptional way through cleavage of the target mRNA or translational repression [56]. Steroid hormones, such as testosterone, have been identified as potential modulators of small RNA activity [57]. Moreover, the half-life of different RNA species is important to determine the length of time in which these mRNA molecules will act as template for protein translation. For instance, Saceda et al [58] showed that P4 promotes the increase in the half-life of its own receptor's mRNA from six to twelve hours, in primary cell culture of endometrial fibroblasts. Presently, studies are being developed in our laboratory to determine whether E2 and P4 alter the mRNA stability of secreted proteoglycans of the mouse uterus.

## Conclusions

This and a previous study from our group suggest that hormonal regulation of mRNA splicing, transcription and post-transcriptional modifications, protein deposition in the ECM, and regulation of metalloproteinases are important events to be considered when studying the differential expression of ECM components, as well as tissue remodeling, in the reproductive organs during the sexual cycle and pregnancy.

## Competing interests

The authors declare that they have no competing interests.

## Authors' contributions

RS carried out the collection and preparation of samples, the staining procedures, the molecular biology experiments and drafted the manuscript. RF participated in the light microscopy experiments, has made substantial contributions to analysis and interpretation of data, and helped to draft the manuscript. TZ conceived of the study, participated in its design and coordination and helped to draft the manuscript. All authors read and approved the final manuscript.
